# RAPID DIGITAL TRANSFORMATION EXPERIENCES FOR SOCIAL CONNECTEDNESS AMONG LOW-INCOME OLDER ADULTS

**DOI:** 10.1093/geroni/igad104.0821

**Published:** 2023-12-21

**Authors:** Jane Chung, Tracey Gendron, Jodi Winship, Rachel Wood, Natalie Mansion, Pamela Parsons

**Affiliations:** Virginia Commonwealth University, Richmond, Virginia, United States; Virginia Commonwealth University, Richmond, Virginia, United States; Virginia Center on Aging, Virginia Commonwealth University, Richmond, Virginia, United States; Bon Secours Mercy Health System, Cincinnati, Ohio, United States; Virginia Commonwealth University College of Health Professions, Richmond, Virginia, United States; Virginia Commonwealth University, Richmond, Virginia, United States

## Abstract

Digital technology has become critical for making social connections and providing social support during COVID-19. However, social isolation challenges disproportionately affected older adults living alone in low-income communities due to a lack of internet access, digital technology ownership, and limited capital. We conducted a mixed-methods study to examine the role of digital technology in this population to keep socially connected during the national lockdown (N=25; 76% African American). Of the participants, 36% reported social isolation, and 36% reported loneliness. Both quantitative and qualitative data illustrate differential experiences of technology use for remaining socially connected. The qualitative data from focus group interviews show that participants were forced to adapt to the isolated situation quickly by learning about new ways of connecting with others through technology. This enabled a long-overdue adoption of digital technologies among individuals who had experienced digital exclusion before the pandemic. Despite the high interest in digital technology-enabled social connections, concerns about its surveilling nature, the possibility of over-dependence on technology, and negative technology experiences due to unreliable functions or lack of tech support made them hesitant to adopt new technology. Future research should highlight the need to include older adults in all stages of technological solution development that recognize the sociocultural context of use with a focus on growth-oriented opportunities for older people.

